# Editorial: Cancer treatment-related cardiovascular disease - real world data in cardio-oncology

**DOI:** 10.3389/fonc.2023.1277042

**Published:** 2023-09-20

**Authors:** Vivek Agarwala, Arjun Ghosh, Avirup Guha, Purvish M. Parikh, Susan Dent

**Affiliations:** ^1^ Medical Oncology & Hemat-Oncology, Narayana Superspeciality Hospital & Cancer Institute, Howrah and RN Tagore International Institute of Cardiac Sciences (RTIICS), Kolkata, India; ^2^ Cardiology, Barts Heart Centre and University College London Hospital, London, United Kingdom; ^3^ Medical College of Georgia, Augusta University, Augusta, GA, United States; ^4^ Clinical Hematology, Mahatma Gandhi University of Medical Sciences Technology, Jaipur, India; ^5^ Duke Cancer Institute, School of Medicine, Duke University, Durham, NC, United States

**Keywords:** cardiotoxicity, CTR-CVT, cardiovascular surveillance, cardiovascular risk stratification, ICI cardiotoxicity, VEGF inhibitor, QTc prolongation, tako tsubo syndrome

Modern anti-cancer therapies have revolutionized cancer treatment. With improving outcomes and survival and cure rates, the significance of cancer treatment-related cardiovascular disease (CTRCD) or toxicity (CTR-CVT) is also being realized (1, 2). The crux of cardio-oncology is to understand such interactions better, to reduce their morbidity and mortality, and to develop protocols and guidelines to manage CTRCD through collaborative efforts in the clinic and research (2, 3). The Global Cardio-oncology Registry has initiated multinational collaboration and has prioritized research on CTRCD in the setting of breast cancer, hematological cancers, and immune-checkpoint inhibitors (ICI) (4). The spectrum of CTRCD, however, is not limited to these alone. Recently published ESC guidelines focus on the definition and management of the entire spectrum of CTRCD (5).

Multiple myeloma is mostly diagnosed in elderly patients with pre-existing cardiovascular (CV) comorbidities (5, 6). Anti-myeloma combination therapy, including immunomodulators (IMiDs), dexamethasone, proteasome inhibitors, and monoclonal antibodies (MABs), has demonstrated an increased risk of serious cardiovascular adverse events (CVAEs), necessitating simple and quick clinical tools for risk stratification (5). Yuan et al., in a retrospective study of 253 newly diagnosed multiple myeloma (NDMM) patients, developed and validated a risk score prognostic and predictive model for CVAEs in this patient population. Patients were divided into training and validation cohorts randomly. Univariate and multivariate analysis identified three independent factors associated with CVAEs – age > 61 years, high baseline blood pressure (BP), and left ventricular (LV) hypertrophy. Age was assigned 2 points and others 1 point each to construct a prognostic model that differentiated NDMM patients into three risk groups - 3–4 points, high risk; 2 points, intermediate risk; 0–1 point, low risk. These groups exhibited significant differences in 1-year and 2-year CVAEs in both the training and validation cohorts. Statistical analysis using C-index values, ROC curves, and decision curve analysis showed that this model had good calibration and provided greater net benefit than the default strategies of CV risk assessment for all patients. This study successfully showed that NDMM patients at increased risk of CVAEs can be identified at baseline, permitting the introduction of CV protective strategies. This model merits further validation in a large prospective cohort for its widespread application and acceptability.

Allogeneic hematopoietic cell transplantation (allo-HCT) has significantly improved cure rates in relapsed refractory hematological malignancies. While the associated long-term impact of increasing CV morbidity and mortality amongst survivors is well known, the short-term CV effects are less studied (7). Dillon et al., in a 3-month prospective study, evaluated the short-term CV impact of allo-HCT in 17 high-risk hematological cancer patients compared to an age-matched non-cancer control group of 12. This was done through pre- and post-transplant cardio-pulmonary exercise testing, to quantify the VO2peak, along with exercise cardiac MRI (for cardiac reserve), resting echocardiography (ECHO), dual-energy x-ray absorptiometry (for lean and fat mass), BP assessment, hemoglobin sampling, and arterio-venous oxygen difference estimation via Fick’s equation. They found significant reductions in the absolute VO2peak, body weight-indexed VO2peak, lean mass, and cardiac reserve in the allo-HCT group, indicating rapid CV aging in this population and the need for early preventive measures. This study also increases our awareness of these early changes and raises the question of whether we should be searching for them in clinical practice.

Hubbert et al. evaluated the long-term incidence, risks, and predictors of CTR-CVT and all-cause mortality in a retrospective Swedish registry study, which included 433 lymph node-positive early breast cancer (EBC) patients considered for adjuvant chemotherapy, who were aged 18-60 years and diagnosed between 1998 and 2002. The CTR-CVT events included hypertension (HT), coronary artery disease (CAD), heart failure (HF), or atrial fibrillation (AF) after the diagnosis of BC. Patients were followed until November 2021 or death. A total of 910 CTR-CVT events were diagnosed in 311/433 women (71.8%), with a median of 19.3 years follow-up - HT 281 (64%); CAD 198 (46%); HF 206 (47%); and AF 225 (51%). Older age (51-60 years) and anthracycline exposure increased the risk of CTR-CVT. Among CTR-CVTs, HF showed the strongest association with anthracycline use (HR 2.0), followed by CAD (HR 1.7) and AF (HR 1.5). At the end of the 24-year study period, 227 of the 433 women were alive, with a 47.6% cumulative mortality. The study demonstrated the high prevalence of CTR-CVT and all-cause mortality after BC diagnosis and treatment, particularly in older patients and those receiving anthracyclines. These findings support the need for CV risk stratification prior to starting anti-cancer therapy and long-term annual screening for CV risk factors and CTR-CVT among BC survivors.

Wu et al. conducted a retrospective study of 2060 consecutive patients with stage 0 – III BC undergoing pre-treatment ECHO (n=1032) or MUGA (n=1028) from 2010 to 2019 at a tertiary cancer care center in Canada. The primary end point was a composite of all-cause mortality and incidence of HF. Follow-up cardiac imaging scans were obtained in 39.3% of patients with MUGA and 38.0% with ECHO. At a median follow-up of 6.7 years, there were 194 deaths, including 7 CV deaths, and 28 heart failure events, with no difference in events between the MUGA and ECHO groups. Patients without follow-up imaging had a similar adjusted risk for the composite outcome compared to those with imaging follow-up, with a hazard ratio of 0.8 (95% CI 0.5-1.3, p=0.457). This study demonstrated that CV deaths and HF event rates were low in a real-world setting and uninfluenced by follow-up CV imaging. Further research is needed to determine the potential benefit of CV imaging surveillance in high-risk patients who have completed anti-cancer therapy.

Head and neck squamous cell carcinoma (HNSCC) patients have a high incidence of CVD and stroke due to common CV risk factors (e.g., tobacco) and cancer treatment-related risk factors such as neck radiotherapy (RT), platinum chemotherapy, and neck surgery (8). Mukherjee et al. assessed the association of HNSCC-related factors [subsite, stage, treatment, human papillomavirus (HPV) status] and traditional CV risk factors [HT, diabetes, dyslipidemia, tobacco, obesity] with 1- and 5-year CVD risk (CAD, ischemic stroke, HF) in a retrospective cohort of 1829 HNSCC patients. Patients treated with RT, HPV +, diabetes, and older age were reported to have a higher risk of CVD. The use of anti-hypertensives at baseline significantly reduced the 1-year and 5-year risk of CVD. The findings are similar to a recently published cohort study of 35897 HNSCC patients that had a high incidence of CV events and HT as the most prevalent CV risk factor (9). These studies highlight the importance of risk-directed primary preventive measures in HNSCC patients to reduce the incidence of CVAEs.

ICIs have improved the clinical outcomes of several types of early and advanced cancer. However, they can cause non-specific activation of the immune system, leading to immune-related adverse events (IRAEs). Moreover, the CV system is not spared. Zhang et al. prospectively analyzed the incidence of CVAEs in 106 ICI-treated cancer patients in a single-center study from China. They found that 38% of patients developed various ECG abnormalities, 36% LV diastolic dysfunction, and 8% LV ejection fraction (LVEF) decline, while 8% saw increased cardiac biomarkers and 2% pericardial effusion. Baseline HT and lower peak early diastolic mitral annulus velocity (e’) predicted a higher incidence of LV dysfunction in ICI-treated patients. Others have reported a higher risk of myocarditis, stress cardiomyopathy, and even an increased risk of atherosclerosis (5). ICI-associated myocarditis, though rare, can often occur early with high fatality (30-50%). Such eventualities may be higher in patients with pre-existing cardiac disease. Hence, a high index of suspicion is required. Put together, there is increasing evidence that the CV IRAEs are likely underestimated. Besides regular CV monitoring including ECG, ECHO, and cardiac biomarkers, cardio-oncologists will have to identify and utilize novel strategies to prevent such acute and long-term sequelae.

Vascular Endothelial Growth Factor (VEGF)-targeting MABs and oral tyrosine kinase inhibitors (TKIs) have shown remarkable efficacy in a variety of cancers, but their associated CVAEs remain poorly elucidated in a real-world setting. Wang et al. looked at the CV toxicity profile associated with all VEGF inhibitors in a comprehensive pharmacovigilance analysis of the FDA Adverse Event Reporting System (FAERS) database from 2014 to 2021. Bevacizumab was reported to have the highest CVAEs (31.8%), followed by Sunitinib (12.4%), amongst all VEGF inhibitors. Amongst CVAEs, HT showed the strongest association, with the shortest time to onset (median 23 days) but the least proportion of death or life-threatening events, whereas thromboembolism had the longest time to onset (median 51 days) but the highest risk of fatality.

QTc prolongation, a known risk factor for fatal ventricular arrhythmias, is seen in 2.5 – 12.5% of patients on anti-cancer treatments, more specifically due to oral TKIs, arsenic trioxide, anti-emetics, and antimicrobials, with some having black-box warnings (10). Risk stratification scores for QTc prolongation have been developed, but they are more applicable to the inpatient and acute care setting. There is a paucity of real-world data in the ambulatory oncology setting. Reeves et al. conducted a single-center retrospective study to describe the incidence and risk factors of QTc prolongation in 49 outpatients on oral TKIs with available baseline and follow-up ECGs. They found a 24% incidence, with 3 patients (6%) having significant QTc prolongation (Bazett formula) - QTc >500 ms or >60 ms increase from baseline. These patients, however, remained asymptomatic, and there was no discontinuation of TKI. Concomitant therapy with a loop diuretic (41% vs 11%, p=0.029) was a risk factor for QTc prolongation. This study showed that real-world incidence of QTc prolongation is higher, and frequent ECG and electrolyte monitoring is needed for patients on oral TKIs, especially those on concomitant loop diuretics.

Takotsubo Syndrome (TTS), a transient and reversible LV systolic dysfunction due to different specific triggers, is known to be more common among cancer patients. However, the relationship between its triggers and cancer outcomes is not well studied. Safdar et al. published a retrospective study from the MD Anderson Cancer Center to determine whether different triggering events for TTS—”cancer-specific triggers” (e.g., chemotherapy, immunomodulators, or RT) or “traditional triggers” (e.g., medical, procedural, and emotional stress)—modified outcomes in 81 identified patients with TTS among 373 cancer patients presenting with acute coronary syndrome over a period of 12 years. This study showed a high prevalence of TTS in cancer patients. A total of 47 out of the 81 TTS patients died, all of these being cancer-related deaths, and there was no CV mortality, with a median survival of 11.9 months. Immunomodulator and RT-related TTS showed higher mortality. Medical triggers showed the least recovery in LVEF and global longitudinal strain (GLS), while patients with emotional and chemotherapy triggers showed the most improvement.

This Research Topic covers a wide spectrum of CTR-CVT and fulfills its aim of collecting meaningful and impactful real-world data in this field.

## Author contributions

VA: Conceptualization, Data curation, Methodology, Project administration, Supervision, Writing – original draft, Writing – review & editing. AGh: Writing – review & editing. AGu: Writing – review & editing. PP: Writing – review & editing. SD: Writing – review & editing.
